# Association between the first 24 hours PaCO_2_ and all-cause mortality of patients suffering from sepsis-associated encephalopathy after ICU admission: A retrospective study

**DOI:** 10.1371/journal.pone.0293256

**Published:** 2023-10-24

**Authors:** Honglian Luo, Gang Li, Bingxin Yang, Xinlei Huang, Yan Chen, Wei Shen

**Affiliations:** 1 Department of Neurology, Puai Hospital of Tongji Medical College, Huazhong University of Science and Technology, Wuhan, Hubei, China; 2 Wuhan Fourth Hospital, Wuhan, Hubei, China; Jinan University First Affiliated Hospital, CHINA

## Abstract

**Objective:**

The relationship between the levels of the first 24-h PaCO_2_ and the prognosis of sepsis-associated encephalopathy (SAE) remains unclear, and the first 24-h optimal target for PaCO_2_ is currently inconclusive. This study was performed to investigate the correlation between PaCO_2_ and all-cause mortality for SAE patients, establish a reference range of the initial 24-hour PaCO_2_ for clinicians in critical care, and explain the possible pathophysiological mechanisms of abnormal PaCO_2_ levels as a higher mortality risk factor for SAE.

**Methods:**

The baseline information and clinical data of patients were extracted from the fourth edition Medical Information Mart for Intensive Care database (MIMIC-IV 2.0). Multivariate logistic regressions were performed to assess the relationship between PaCO_2_ and all-cause mortality of SAE. Additionally, restricted cubic splines, Kaplan-Meier Survival analyses, propensity score matching (PSM) analyses, and subgroup analyses were conducted.

**Results:**

A total of 5471 patients were included in our cohort. In the original and matched cohort, multivariate logistic regression analysis showed that normocapnia and mild hypercapnia may be associated with a more favorable prognosis of SAE patients, and survival analysis supported the findings. In addition, a U-shaped association emerged when examining the initial 24-hour PaCO_2_ levels in relation to 30-day, 60-day, and 90-day mortality using restricted cubic splines, with an average cut-off value of 36.3mmHg (*P* for nonlinearity<0.05). Below the cut-off value, higher PaCO_2_ was associated with lower all-cause mortality, while above the cut-off value, higher PaCO_2_ was associated with higher all-cause mortality. Subsequent subgroup analyses revealed similar results for the subcohort of GCS≤8 compared to the original cohort. Additionally, when examining the subcohort of GCS>8, a L-shaped relationship between PaCO2 and the three clinical endpoints emerged, in contrast to the previously observed U-shaped pattern. The findings from the subcohort of GCS>8 suggested that patients experiencing hypocapnia had a more unfavorable prognosis, which aligns with the results obtained from corresponding multivariate logistic regression analyses.

**Conclusion:**

The retrospective study revealed the association between the first 24-h PaCO_2_ and all-cause mortality risk (30-day, 60-day, and 90-day) for patients with SAE in ICU. The range (35mmHg-50mmHg) of PaCO_2_ may be the optimal target for patients with SAE in clinical practice.

## 1. Introduction

Sepsis is a life-threatening organ dysfunction, which occurs in the course of the host’s uncontrolled inflammation response to infection [1]. The presence of systemic inflammation can disrupt the delicate balance of the normal physiology of the central nervous system (CNS), leading to acute neurological dysfunction that may manifest as sepsis-associated encephalopathy (SAE) [2].

SAE represents a frequent comorbidity of sepsis, featuring altered neurocognitive status without direct evidence of intracranial infection or other diagnosed encephalopathies [3]. The exact pathogenesis of sepsis-associated encephalopathies (SAE) is not fully elucidated. Several possible mechanisms are being investigated, including microscopic brain injury, blood-brain barrier dysfunction, the inadequate function of cerebral microcirculation, deregulated neurotransmission, and altered brain metabolism [4]. It is crucial to recognize that these potential mechanisms involved in SAE may not act independently. Instead, they interact at various stages of the disease and collectively contribute to SAE onset and progression. Because of lacking the uniform definition, the incidence of SAE varies from 9% to 71%, however, the majority of studies hold that the incidence of SAE is very high [5, 6]. Although the exact mechanisms are not clear, the neurological status of sepsis patients is increasingly recognized as a critical determinant of outcomes. SAE can significantly worsen the prognosis for sepsis patients, impacting their recovery and survival. The result of a cohort study confirmed a strong association between the severity of SAE and hospital mortality [7]. The diagnosis of SAE is still a challenge. It relies on clinical evaluation and the exclusion of other possible causes of altered mental status, which may resulting in delayed diagnosis and exacerbation of the condition. The modifiable factors that could potentially contribute to the mortality of sepsis-associated encephalopathy are not entirely elucidated. Therefore, investigating the factors that may influence the prognosis of SAE patients is clinically significant, which may be a potentially effective management strategy in clinical practice.

Adequate cerebral perfusion is essential for maintaining normal brain function. A variety of mechanisms tightly regulate cerebral blood flow (CBF) to meet the high metabolism rate of the brain [3]. However, cerebral ischemia has been confirmed in patients with sepsis and different areas of the brain can find ischemic lesions [8]. Impaired autoregulation and reduced CBF may play a pivotal role in the development of sepsis-associated encephalopathy [9].

It is well-established that CO_2_ is a potent regulator of the cerebral vascular tone. However, the role of PaCO_2_ for SAE is unclear. The association between PaCO_2_ and mortality, as well as the optimal targets of PaCO_2_ in patients with SAE, remain poorly defined. For this purpose, we performed a retrospective study to explore the relationship between PaCO_2_ and mortality in SAE patients, as well as identify the optimal range of PaCO_2_ to provide reference to critical care clinicians in managing sepsis patients with SAE.

## 2. Methods

### 2.1 Database

This retrospective study utilized de-identified data from the medical information mart for intensive care (MIMIC IV 2.0) [10]. The database is large, open-source, and freely accessible, which contains 76943 ICU admissions of patients admitted by critical care units at the Beth Israel Deaconess Medical Center (BIDMC, Boston, MA, USA) between 2008 and 2019. One of the authors (Honglian Luo) successfully fulfilled the requirement of the web course on “protecting human research participants”, passed the corresponding tests, and obtained authorization to make use of the public data for scientific research (certificate number 53403674). Establishment of the MIMIC database was approved by the Massachusetts Institute of Technology (Cambridge, MA) and Beth Israed Deaconess Medical Center (Boston, MA) (No.27653720), and since the data in the MIMIC database is anonymous, the ethical review was waived by the Institutional Review Boards of BIDMC.

We have completed all items on the Clinical studies Checklist [11] and the manuscript complied with the Strengthening the Reporting of Observational Studies in Epidemiology (STROBE) Statement [12].

### 2.2 Study population and variable extraction

#### 2.2.1 Population

**Fig 1:**
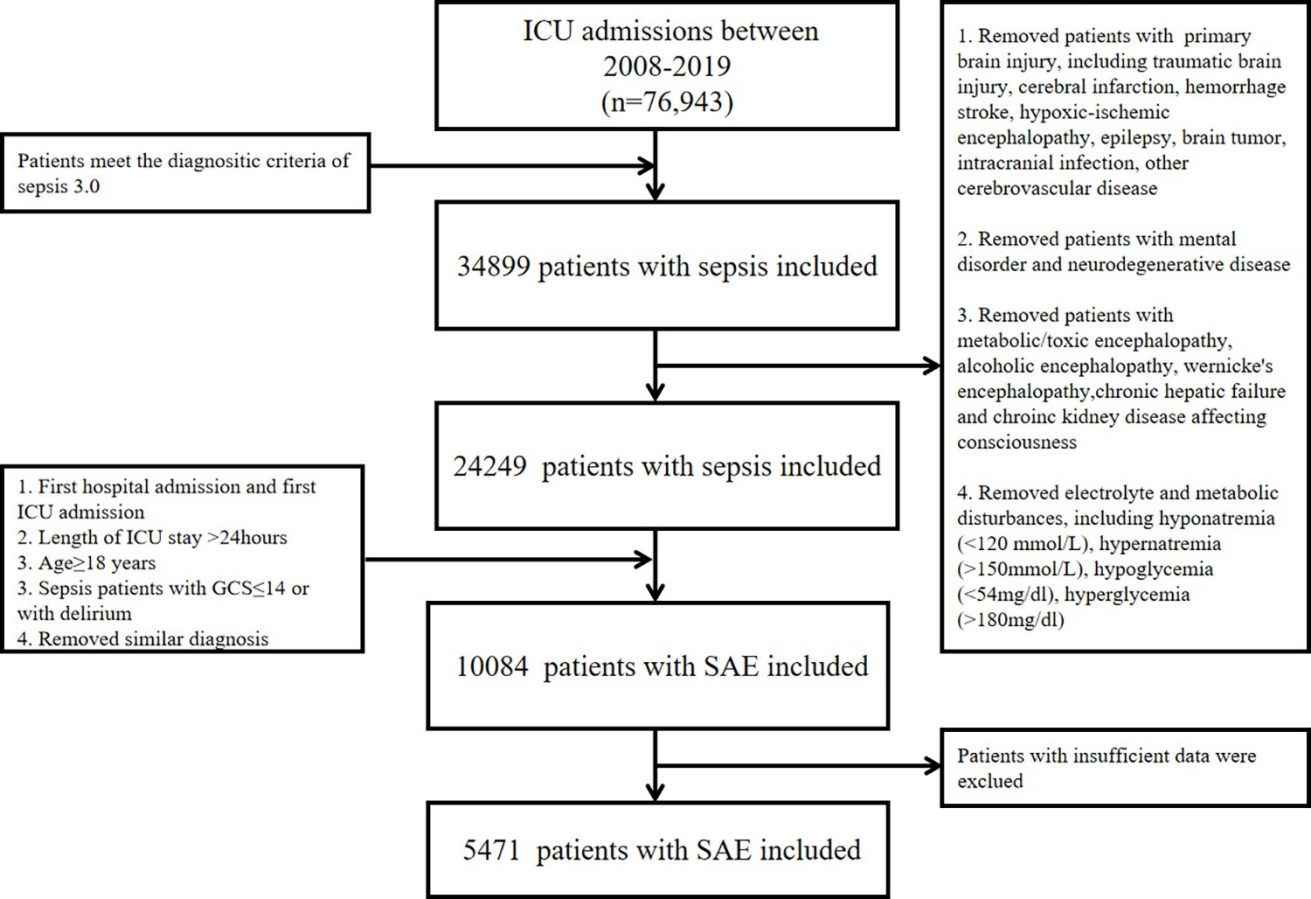
Flow chart of study cohort selection process. ICU, intensive care unit. SAE, sepsis-associated encephalopathy.

Patients with sepsis were defined based on the third edition of the sepsis diagnostic criteria (Sepsis 3.0), which combines sequential organ failure assessment score (SOFA≥2) and suspected or confirmed infection [1]. Sepsis-associated encephalopathy (SAE) is characterized by altered mental status. Diagnosis of SAE relies on clinical evaluation and the exclusion of other possible causes of altered mental status. In the study, the change of mental status referred to GCS ≤14 or delirium. Specifically, the inclusion criteria were as follows: (1) patients complied with the diagnostic criteria of Sepsis 3.0; (2) only data for the first ICU stay were collected for patients; (3) Length of ICU stay ≥24 hours; (4) age≥18 years; (5) sepsis patients with GCS ≤14 or with delirium. The exclusion criteria were: (1) patients suffering from primary brain injury, including traumatic brain injury, cerebral infarction, intracranial hemorrhage, hypoxic-ischemic encephalopathy, epilepsy, brain tumor, intracranial infection, and other encephalopathies with a definite diagnosis; (2) patients suffering from mental disorders and neurodegenerative disease; (3) patients suffering from metabolic/toxic encephalopathies, alcoholic encephalopathy, Wernicke’s encephalopathy, chronic hepatic failure altering mental status, and chronic kidney disease affecting consciousness; (4) patients with electrolyte and metabolic disturbances, included hypernatremia (>150mmol/L), hyponatremia (<120mmol/L), hyperglycemia (>180mg/dl), hypoglycemia (<54mg/dl); (5) Patients with insufficient data were excluded. Fig 1 recorded the selection process for the study cohort.

#### 2.2.2 Variable extraction

The baseline information and clinical data were retrieved, collected, and processed using Navicat Premium (version 15.0.12). The following first-24h variables of ICU admission were extracted from the MIMIC-IV database (version 2.0), including age, gender, Glasgow Coma Scale (GCS), and sequential organ failure assessment score (SOFA). Laboratory variables included PaCO_2,_ sodium, potassium, bicarbonate, chloride, blood urea nitrogen (BUN), serum creatinine (sCr), partial thromboplastin (PTT), hemoglobin (Hb), mean corpuscular hemoglobin (MCH), platelet, white blood cell (WBC), lactate, base excess (BE) and glucose (Glu). Vital signs included temperature (Tem), heart rate, systolic blood pressure, diastolic blood pressure, and pulse oxygen saturation (SpO_2_). Comorbidities included myocardial infarction (MI), congestive heart failure (CHF), chronic pulmonary disease (CPD), diabetes (DM), hypertension (HTN), chronic kidney disease (CKD), cancer, and liver disease, acute kidney injury (AKI). Treatment included first-day ventilation, first-day RRT, vasoactive medication, and diuretics. On the first day of admission, if a variable was evaluated more than once, use the average result.

The clinical endpoint in the study was 30-day, 60-day, 90-day mortality after ICU admission.

### 2.3 Statistical analysis

Our study is a retrospective analysis that utilized data extracted from MIMIC IV 2.0. Therefore, we did not conduct a sample size estimation for this study. The Kolmogorov- Smirnov (KS) test was conducted to analyze the normality of data distribution for continuous variables. Because all of the continuous variables in our investigation were not normally distributed, values were described as medians [interquartile ranges (IQRs)]. All categorical variables were reported as the count (percentage). Continuous values were compared using a non-parametric test (Mann-Whitney U test) and categorical variables were analyzed by chi-square test between two groups (survivors VS non-survivors). Univariate binary logistic regression analysis was carried out to identify possible independent risk factors (*P* value<0.05). In addition, a collinearity diagnostic test was performed to check for collinearity, and variables whose variable inflation factors (VIF) were >10 were removed from the logistic regression model (S1 Table in S1 File). Then, multivariate binary logistic regression analyses (forward method, step-by-step selection) were conducted to identify the independent risk factors for 30-day mortality after ICU admission and explore the relationship between PaCO_2_ and all-cause mortality risk in patients with SAE. All the results of regression analyses were reported as odds ratios (ORs), 95% confidence intervals (CIs), and *P* values. Considering the nonlinear association between PaCO_2_ and all-cause mortality risk, restricted cubic splines (RCS) employing logistic regression, with four knots at the 5th, 35th, 65th, and 95th centiles, was applied to model the association of PaCO2 with mortality risk of patients with SAE. All covariates on the first day of ICU admission were adjusted in the spline models for the original cohort.

To enhance the credibility of the results in the original cohort, we conducted a propensity score matching (PSM) between two groups, i.e., death and survival, as a sensitivity analysis. Potential confounding factors were incorporated in the propensity score analysis (except for GCS). A 1:1 nearest neighbor matching algorithm (with a caliper of 0.03) was used, and no replacement was allowed. The propensity score was calculated using a logistic regression model. A comparison of the standardized mean difference (SMD) in the original and matched cohorts was performed to evaluate the effectiveness of PSM. We employed the same analytical methods, including multivariate binary logistic regression and restricted cubic splines regressions, to analyze the matched cohort, and GCS, a unbalanced covariate in the matched cohort, was adjusted.

Based on the results of multivariate binary logistic regression analyses and restricted cubic splines analysis, both the original and matched cohort were divided into three groups (<35mmHg, 35-50mmHg, and ≥50mmHg, respectively). The Kaplan-Meier Survival curves were plotted, and differences were evaluated among groups by Log-rank tests. Additionally, a subgroup analysis by stratifying the original cohort based on the level of disturbance of consciousness, was performed using similar methods as previously described.

The statistical analysis was performed by using SPSS (version 25.0) software and R (version 4.2.1). A *P*-value < 0.05 was defined as statistically significant.

## 3. Results

### 3.1 Baseline characteristics

**Fig 2:**
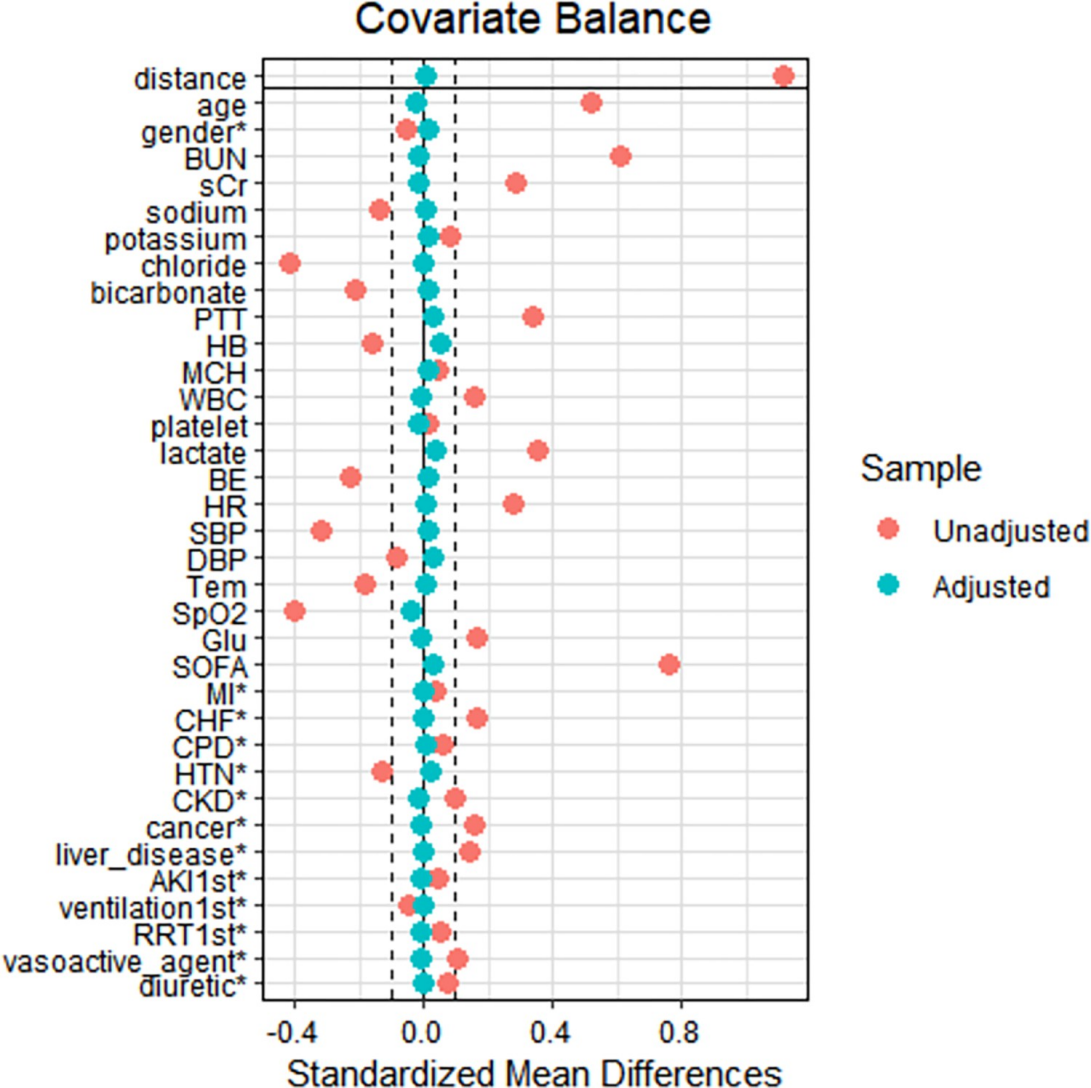
The comparison of SMDs in the original and matched cohorts. SMD, standardized mean difference.

After screening the data of 76943 ICU admissions, a total of 5471 patients were ultimately identified from the MIMIC-IV database (Table 1). Patients in our cohort were divided into two groups based on their survival outcome [survival (4617, 84.4%) vs. death (854, 15.6%)]. The median age of non-survivors was significantly higher than survivors (75.0 vs. 67.0, *P*<0.001). The median SOFA score in the non-survival group was higher than in the survival group (10.0 vs. 7.0, *P*<0.001), while the median GCS score in the non-survival group was lower than those in the survival group (9.0 vs. 13.0, *P*<0.001). The other continuous covariates, except for potassium (3.9 vs. 3.9, *P* = 0.174) and platelet (171.5 vs. 172.5, *P* = 0.054), differed significantly between the death group and the survival group (*P*<0.05). For categorical variables, the proportions of receiving RRT, vasoactive and diuretic medication of ICU admission were significantly higher in the death group than in the survival group (*P*<0.001). In addition, the patients in the death group had a higher rate of comorbidities (MI, CHF, CPD, CKD, cancer, liver disease, and first-day AKI) (*P*<0.001 for the first six covariates, *P* = 0.01 for first-day AKI, respectively). Propensity matching score analysis adjusted all potential confounders except for GCS. The reason for excluding GCS is that incorporating GCS resulted in too many death samples losing, which would lower the credibility of the matched cohort. After performing propensity score matching (PSM) to adjust for all potential confounders (except for GCS), the SMDs in the matched cohort ranged from -1~1. The corresponding covariates, except for GCS (*P*<0.001), were more balanced and comparable between the two groups (*P*>0.05) (Fig 2, Table 1). It is worth noting that the PaCO_2_ had statistically significant differences between the survival and death groups, regardless of whether propensity score matching was performed. Other clinical characteristics of SAE patients were described in Table 1.

**Table 1:** Baseline clinical data of the study population.

	Original cohort			Matched cohort		
Variables	Survivors N (4617)	Non-survivors N (854)	*P*-value	Survivors N (725)	Non-survivors N (725)	*P*-value
Age (year)	67.0[57.0, 77.0]	75.0[63.0, 84.0]	<0.001	75.0[64.0, 83.0]	74.0[62.5, 84.0]	0.867
Male, n (%)	2748(59.5%)	460(53.9%)	0.002	387(29.5%)	397(29.8%)	0.598
**PaCO**_**2**_ **(mmHg), n (%)**						
<30	340(7.4%)	129(15.1%)	<0.001	93(12.8%)	107(14.8%)	0.023
30–35	902(19.2%)	188(22.0%)		139(19.2%)	160(22.1%)	
35–45	374(8.1%)	82(9.6%)		65(9.0%)	75(10.3%)	
45–50	341(7.4%)	105(12.3%)		71(9.8%)	89(12.3%)	
≥50	2660(57.6%)	350(41.0%)		357(49.2%)	294(40.6%)	
**Scoring systems**						
GCS	13.0[9.0, 14.0]	9.0[3.0, 13.0]	<0.001	12.0[8.0, 14.0]	10.0[3.0, 13.0]	<0.001
SOFA	7.0[5.0, 9.0]	10.0[7.0, 14.0]	<0.001	10.0[7.0, 12.0]	9.0[7.0, 13.0]	0.744
**Vital signs**						
Temperature (°C)	36.9[36.6, 37.2]	36.8[36.4, 37.1]	<0.001	36.8[36.5, 37.1]	36.8[36.5, 37.1]	0.870
Heart Rate (bpm)	84.8[16.6, 96.1]	91.0[79.5, 103.6]	<0.001	90.0[79.2, 101.9]	90.1[78.4, 102.9]	0.718
Systolic BP (mmHg)	112.4 [105.5, 120.3]	108.0 [100.5, 117.0]	<0.001	108.9 [102.5, 117.4]	108.6 [101.0, 118.0]	0.615
Diastolic BP (mmHg)	58.9[53.8, 64.6]	57.9[51.9, 64.5]	0.004	58.1[53.0, 64.6]	58.3[52.3, 65.0]	0.942
SpO_2_ (%)	97.6[96.3, 98.7]	96.7[94.9, 98.2]	<0.001	96.8[95.3, 98.1]	96.8[95.0, 98.3]	0.765
**Laboratory test**						
Sodium (mmol/L)	138.5 [136.5, 140.0]	138.0 [134.5, 141.0]	<0.001	138.0 [135.5, 140.5]	138.0 [135.5,141.0]	0.820
Potassium (mmol/L)	3.9[3.6, 4.3]	3.9[3.5, 4.4]	0.174	3.9[3.5, 4.4]	3.9[3.5, 4.4]	0.951
Bicarbonate (mmol/L)	23.0[20.5, 25.0]	21.5[18.0, 25.0]	<0.001	22.0[18.5, 24.5]	21.5[18.5, 25.0]	0.761
Chloride (mmol/L)	106.0 [102.5, 109.0]	103.0[98.5, 107.0]	<0.001	104.0 [99.0, 107.5]	103.5 [99.0, 107.5]	0.858
BUN (mmol/L)	18.5[13.5, 28.0]	33.5[20.5, 52.0]	<0.001	29.0[18.5, 47.5]	31.5[20.0, 47.5]	0.267
Creatinine (mg/dL)	1.1[0.9, 1.6]	1.4[1.0, 2.0]	<0.001	1.4[1.0, 2.0]	1.3[1.0, 2.0]	0.525
PTT (s)	31.8[28.0, 39.2]	36.7[29.9, 51.4]	<0.001	34.8[29.5, 49.1]	35.8[29.3, 49.8]	0.497
Hemoglobin (g/dl)	10.4[9.3, 11.6]	10.0[8.8, 11.5]	<0.001	10.1[8.9, 11.3]	10.1[8.8, 11.5]	0.440
MCH (g/dl)	30.4[28.9, 31.6]	30.4[28.9, 31.9]	0.282	30.3[28.9, 31.6]	30.4[28.9, 31.7]	0.849
Platelet (10^9^/L)	172.5 [129.3, 234.3]	171.5 [106.0, 252.0]	0.054	178.0 [121.5, 262.4]	177.0 [114.2, 255.9]	0.331
WBC (10^9^/L)	12.3[9.3, 15.9]	13.3[8.8, 18.6]	<0.001	12.8[9.3, 17.7]	12.9[8.75, 18.1]	0.989
Lactate (mmol/L)	1.8[1.3, 2.5]	2.2[1.4, 3.6]	<0.001	2.1[1.4, 3.3]	2.1[1.4, 3.2]	0.805
BE (mmol/L)	-1.0[-3.5, 1.0]	-2.5[-7.0, 1.0]	<0.001	-2.0[-5.8, 0.5]	-2.0[-6.5, 1.0]	0.972
Glucose (mg/dl)	131.1 [118.5, 152.7]	136.6 [114.5, 174.6]	0.006	138.8 [118.4, 173.0]	137.0 [114.8, 176.1]	0.454
**Comorbidities**						
MI, n (%)	896(19.4%)	199(23.3%)	0.009	177(24.4%)	174(24.0%)	0.854
CHF, n (%)	1254(27.2%)	371(43.4%)	<0.001	319(44.0%)	316(43.6%)	0.874
CPD, n (%)	1267(27.4%)	288(33.7%)	<0.001	248(34.2%)	255(35.2%)	0.699
DM, n (%)	1399(30.3%)	241(28.2%)	0.223	225(31.0%)	211(29.1%)	0.423
HTN, n (%)	2174(47.1%)	290(34.0%)	<0.001	229(31.6%)	247(34.1%)	0.314
CKD, n (%)	811(17.6%)	232(27.2%)	<0.001	207(28.6%)	195(26.9%)	0.481
Cancer, n (%)	504(10.9%)	226(26.5%)	<0.001	181(25.0%)	176(24.3%)	0.761
Liver disease, n (%)	545(11.8%)	222(26.0%)	<0.001	166(22.9%)	164(22.6%)	0.900
AKI(1st), n (%)	853(18.5%)	194(22.7%)	0.004	164(22.6%)	158(21.8%)	0.705
**Treatment, n (%)**						
Ventilation(1st), n (%)	3912(84.7%)	682(79.9%)	<0.001	576(79.4%)	577(79.6%)	0.948
RRT(1st), n (%)	178(3.9%)	80(9.4%)	<0.001	69(9.5%)	62(8.6%)	0.521
Vasoactive agent, n (%)	3061(66.3%)	654(76.6%)	<0.001	542(74.8%)	536(73.9%)	0.718
Diuretics, n (%)	1032(22.4%)	257(30.1%)	<0.001	214(29.5%)	216(29.8%)	0.908

GCS, Glasgow Coma Scale; SOFA, Sequential Organ Failure Assessment; BP, blood pressure; SpO_2_, pulse oxygen saturation; BUN, blood urea nitrogen; PTT, partial thromboplastin; MCH, mean corpuscular hemoglobin; WBC, white blood cell; BE, base excess; MI, myocardial infarction; CHF, congestion heart failure; CPD, chronic pulmonary disease; DM, diabetes mellitus; HTN, hypertension; CKD, chronic kidney disease; AKI, acute kidney injury; RRT, renal replacement therapy.

### 3.2 Independent factors of 30-day mortality risk for SAE in the original cohort

As listed in Table 2, We present the results of the multivariate binary logistic regression analyses of 30-day mortality risk for SAE patients after ICU admission. Among variables included in multivariate binary logistic regression analyses, moderate and severe hypocapnia (PaCO_2_<30mmHg) [OR 1.486, 95%CI 1.084–2.038, *P* = 0.014], mild hypocapnia (30mmHg≤PaCO_2_<35mmHg) (OR 1.416, 95% CI 1.120–1.792, *P* = 0.004), as well as moderate-to-severe hypercapnia (PaCO_2_≥50mmHg) (OR 1.439, 95% CI 1.023–2.025, *P* = 0.037) may be potential independent risk factors of 30-day mortality for SAE patients. Furthermore, age (OR 1.057, 95% CI 1.049–1.065, *P*<0.001), SOFA score (OR 1.121, 95% CI 1.085–1.159, *P*<0.001), sodium (OR 1.076, 95% CI 1.042–1.111, P<0.001), BUN (OR 1.014, 95% CI 1.009–1.019, *P*<0.001), PTT (OR 1.009, 95% CI 1.005–1.013, *P*<0.001), platelet (OR 1.001, 95% CI 1.000–1.002, *P* = 0.005), heart rate (OR 1.020, 95% CI 1.014–1.026, *P*<0.001), cancer (OR 2.678, 95% CI 2.148–3.339, *P*<0.001), liver disease (OR 1.973, 95% CI 1.529–2.545, *P*<0.001) had significant effects on the risk of 30-day mortality for SAE patients. Additionally, the other variables shown in Table 2 may be considered protective factors (OR<1, *P*<0.05).

**Table 2:** Multivariate binary logistic regression analysis of risk factors for 30-day all-cause mortality of patients with SAE in ICU.

Variables	OR	95%CI	*P* value
Age	1.057	1.049–1.065	<0.001
GCS	0.881	0.859–0.903	<0.001
SOFA	1.121	1.085–1.159	<0.001
Sodium	1.076	1.042–1.111	<0.001
Bicarbonate	0.957	0.929–0.986	0.003
Chloride	0.892	0.869–0.916	<0.001
BUN	1.014	1.009–1.019	<0.001
Creatinine	0.790	0.700–0.891	<0.001
PTT	1.009	1.005–1.013	<0.001
Hemoglobin	0.939	0.895–0.986	0.011
Platelet	1.001	1.000–1.002	0.005
Temperature	0.728	0.631–0.841	<0.001
Heart Rate	1.020	1.014–1.026	<0.001
Systolic BP	0.983	0.975–0.990	<0.001
SpO_2_	0.868	0.833–0.906	<0.001
PaCO_2_			
35-45mmHg	Ref.	Ref.	Ref.
<30	1.486	1.084–2.038	0.014
30–35	1.416	1.120–1.792	0.004
45–50	1.360	0.991–1.866	0.057
≥50	1.439	1.023–2.025	0.037
DM	0.775	0.635–0.946	0.012
HTN	0.745	0.617–0.900	<0.001
Cancer	2.678	2.148–3.339	<0.001
Liver disease	1.973	1.529–2.545	<0.001
Ventilation(1st)	0.763	0.609–0.957	0.019

Ref, the range (35–45 mmHg) of PaCO_2_ was defined as reference (OR = 1).

### 3.3 The association between PaCO_2_ and the prognosis

We conducted multivariate binary logistic regression analyses to explore the relationship between PaCO_2_ and the prognosis in SAE patients, with the unadjusted and adjusted models shown in Table 3. The unadjusted model 1 showed that hypocapnia (PaCO_2_<35 mmHg) and moderate-to-severe hypercapnia (PaCO2≥50mmHg) were strongly associated with 30-day mortality (OR>1, *P*<0.001). Even after adjusting for all potential covariates, we observed that PaCO_2_<30mmHg, 30mmHg≤PaCO_2_<35mmHg, and PaCO_2_≥50mmHg had a higher risk than 35-45mmHg (OR = 1.486, 95%CI 1.084–2.038, *P* = 0.014; OR = 1.416, 95%CI 1.120–1.792, *P* = 0.004; OR = 1.439, 95%CI 1.023–2.025, *P* = 0.037, respectively), while mild hypercapnia (45≤PaCO_2_<50mmHg) didn’t show significant differences compared with normocapnia (35≤PaCO_2_<45mmHg) (OR = 1.360, 95%CI 0.99–1.866, *P* = 0.057). In order to explore the correlation between PaCO_2_ and longer-term prognosis, 60-day mortality and 90-day mortality were listed as secondary outcomes. Similar to the result of 30-day mortality, both hypocapnia and moderate-to-severe hypercapnia displayed an association with 60-day mortality and 90-mortality, even after controlling for all potential confounding factors. The results from the matched cohort were consistent with the original cohort’s findings (Table 4).

**Table 3:** Two models analysis of the association between PaCO_2_ and all-cause mortality in original cohort.

Original cohort	Logistic Model 1			Logistic Model 2		
Outcomes	OR	95%CI	*P*-value	OR	95%CI	*P*-value
**30-day mortality**						
**35-45mmHg**	**Ref.**			**Ref.**		
<30	2.884	2.288–3.634	<0.001	1.486	1.084–2.038	0.014
30–35	1.584	1.306–1.921	<0.001	1.416	1.120–1.792	0.004
45–50	1.666	1.280–2.169	<0.001	1.360	0.991–1.866	0.057
≥50	2.340	1.831–2.991	<0.001	1.439	1.023–2.025	0.037
**60-day mortality**						
**35-45mmHg**	**Ref.**			**Ref.**		
<30	3.145	2.532–3.908	<0.001	1.683	1.251–2.265	0.001
30–35	1.595	1.334–1.908	<0.001	1.459	1.171–1.818	0.001
45–50	1.635	1.277–2.095	<0.001	1.280	0.948–1.728	0.107
≥50	2.442	1.940–3.073	<0.001	1.498	1.087–2.065	0.014
**90-day mortality**						
**35-45mmHg**	**Ref.**			**Ref.**		
<30	3.151	2.549–3.894	<0.001	1.673	1.252–2.235	<0.001
30–35	1.514	1.272–1.802	<0.001	1.337	1.080–1.656	0.008
45–50	1.613	1.270–2.048	<0.001	1.271	0.950–1.701	0.106
≥50	2.491	1.995–3.113	<0.001	1.613	1.182–2.202	0.003

Ref. the range (35–45 mmHg) of PaCO_2_ was defined as refence (OR = 1); Logistic model 1, not adjusted any covariates; logistic model 2, adjusted for all potential covariates, including age, gender, GCS, SOFA, sodium, potassium, bicarbonate, chloride, BUN, creatinine, PTT, hemoglobin, MCH, platelet, WBC, lactate, and BE, temperature, heart rate, systolic blood pressure, diastolic blood pressure, pulse oxygen saturation, and glucose, myocardial infarction, congestive heart failure, chronic pulmonary disease, diabetes, hypertension, chronic kidney disease, cancer, and liver disease, acute kidney injury, first-day ventilation, first-day RRT, vasoactive medication, and diuretics.

**Table 4:** Two models analysis of the association between PaCO_2_ and all-cause mortality in matched cohort.

Matched cohort	Logistic Model 1			Logistic Model 2		
Outcomes	OR	95%CI	*P*-value	OR	95%CI	*P*-value
**30-day mortality**						
**35-45mmHg**	**Ref.**			**Ref.**		
<30	1.397	1.017–1.920	0.039	1.511	1.092–2.090	0.013
30–35	1.398	1.062–1.840	0.017	1.438	1.086–1.903	0.011
45–50	1.401	0.971–2.021	0.071	1.410	0.971–2.048	0.071
≥50	1.522	1.075–2.156	0.018	1.500	1.052–2.139	0.025
**60-day mortality**						
**35-45mmHg**	**Ref.**			**Ref.**		
<30	1.474	1.069–2.032	0.018	1.593	1.148–2.212	0.005
30–35	1.377	1.045–1.815	0.023	1.414	1.067–1.871	0.016
45–50	1.226	0.850–1.769	0.276	1.227	0.844–1.782	0.284
≥50	1.717	1.203–2.451	0.003	1.699	1.182–2.441	0.004
**90-day mortality**						
**35-45mmHg**	**Ref.**			**Ref.**		
<30	1.456	1.053–2.014	0.023	1.562	1.123–2.173	0.008
30–35	1.350	1.022–1.783	0.034	1.381	1.041–1.831	0.025
45–50	1.225	0.847–1.772	0.281	1.225	0.842–1.782	0.289
≥50	1.802	1.253–2.591	0.001	1.783	1.233–2.579	0.002

All the annotations and instructions in the table are the same as Table 3.

**Fig 3:**
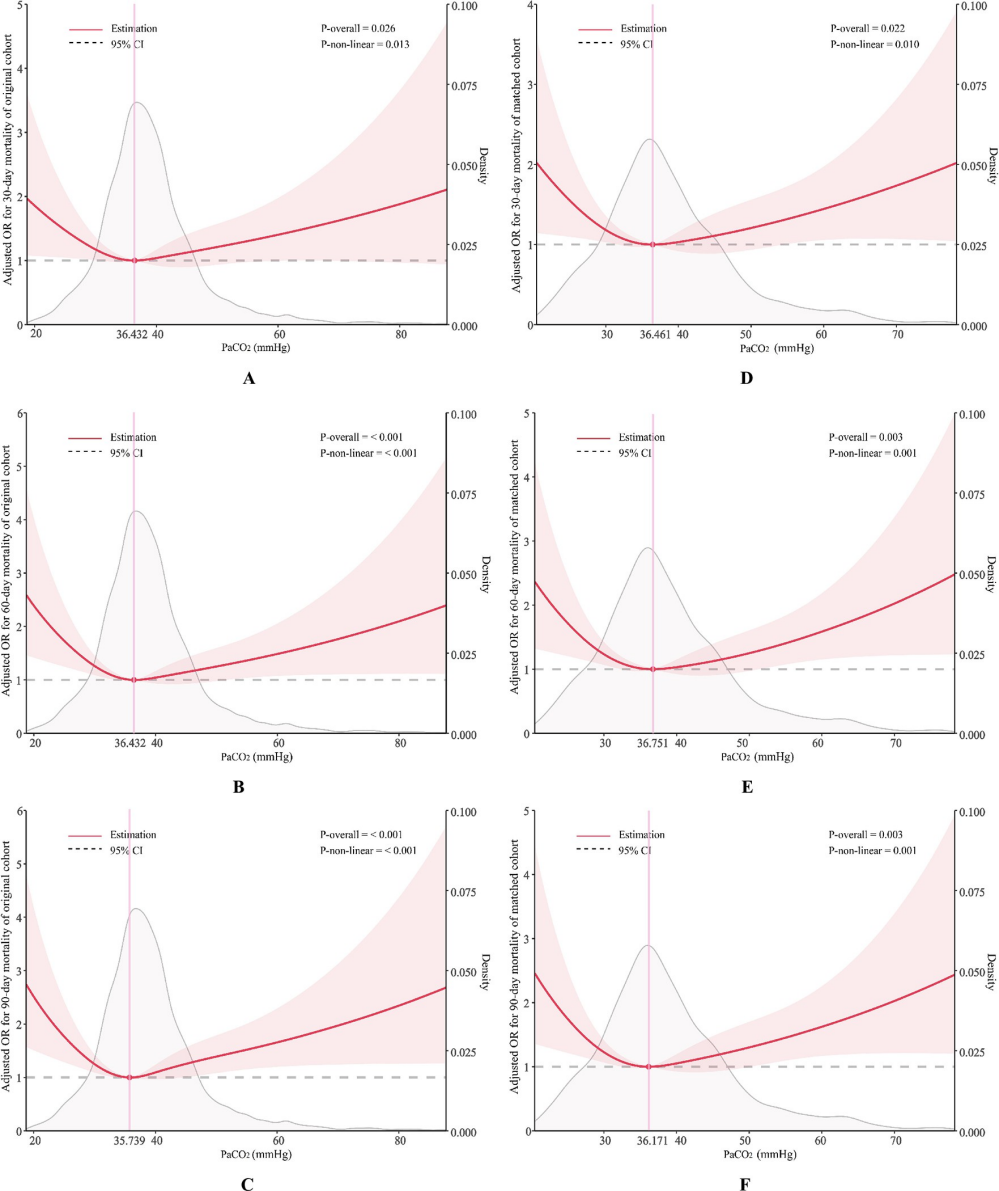
Association of the PaCO2 and the three clinical endpoints in the original and matched cohorts. (A-C) RCS of the original cohort; (D-E) RCS of the matched cohort.

In order to comprehensively investigate potential non-linear relationships, restricted cubic splines (RCS) were employed on a continuous scale to model the association between PaCO_2_ and mortality in patients with SAE, while controlling for all confounding variables. For the original and matched cohorts, the restricted cubic splines analysis showed that the association between PaCO_2_ and 30-day, 60-day, and 90-day mortality of SAE patients was U-shaped (*P* for nonlinearity < 0.05. The nadir of mortality risk was estimated from restricted cubic splines to be approximately 36.3 mmHg (employing the average of nadirs in the original and matched cohorts) (Fig 3).

### 3.4 Comparison of Kaplan-Meier curves

**Fig 4:**
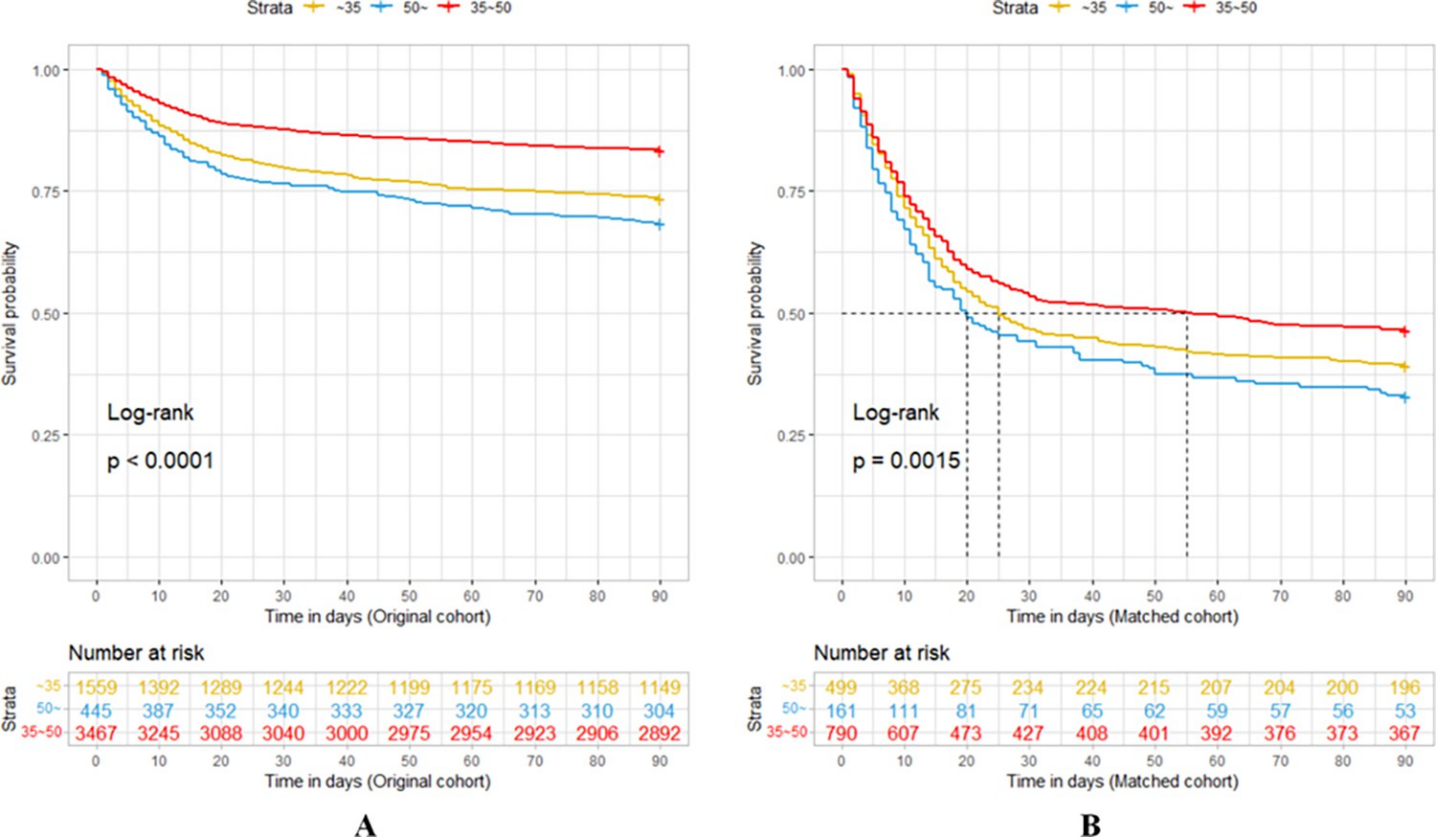
Kaplan-Meier survival curves for SAE patients. SAE, sepsis-associated encephalopathy.

According to the results of multivariate binary logistic regressions and restricted cubic spline regressions, we divided the subjects into three groups based on the PaCO_2_ levels (hypocapnia: PaCO_2_<35mmHg; normocapnia and mild hypercapnia: 35≤PaCO_2_<50mmHg; moderate to severe hypercapnia: PaCO_2_≥50mmHg). The survival of each group was analyzed using the Kaplan-Meier survival curves to examine the effect of PaCO_2_ on the prognoses of SAE patients. In both the original and matched cohorts, the group of normocapnia and mild hypercapnia had the highest survival probability compared to the other groups (Log-rank test: *P*<0.0001; *P* = 0.0015, respectively) (Fig 4). Additionally, in the matched cohort, the normocapnia and mild hypercapnia group had the highest median survival time (55 days), followed by the hypocapnia group (25 days) and then the group of moderate to severe hypercapnia (20 days). In conclusion, the survival curves showed that SAE patients with PaCO_2_<35mmHg or PaCO_2_≥50mmHg had poor prognoses.

### 3.5 Subgroup analysis

Additionally, we conducted a subgroup analysis by stratifying the original cohort based on severe consciousness disorder (GCS≤8) and non-severe consciousness disorder (GCS>8). We utilized multivariate logistic regressions, restricted cubic splines, and the Kaplan-Meier method to examine the association between PaCO_2_ and all-cause mortality in subcohorts of SAE patients.

**Fig 5:**
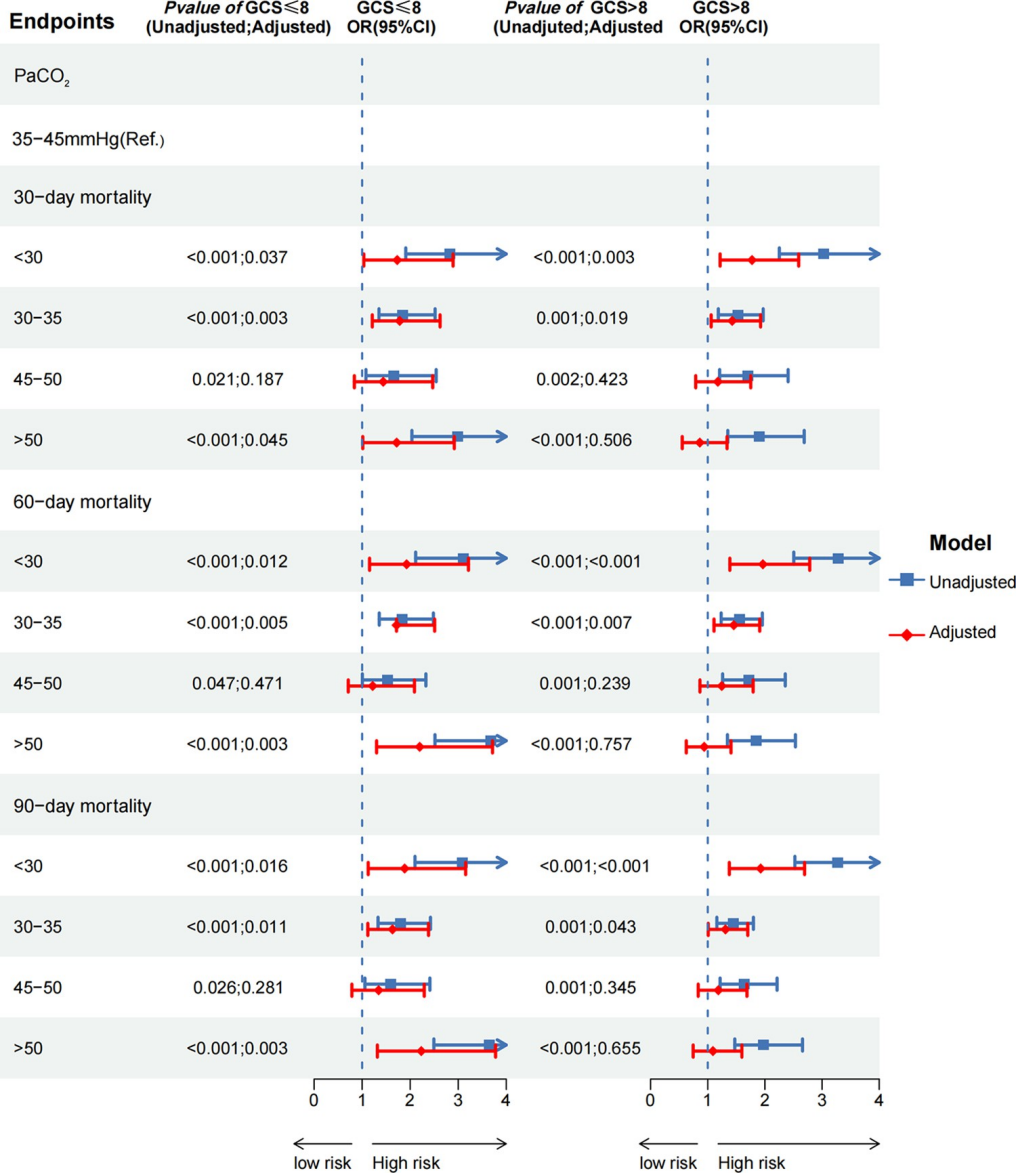
The forest plot showed the correlation between PaCO_2_ and three clinical endpoints in different subcohorts.

In the subcohort of SAE patients with GCS≤8, the findings were similar to those of the original cohort. It was found that hypocapnia (PaCO_2_<35mmHg) and moderate-to-severe hypercapnia (PaCO_2_≥50mmHg) were associated with higher 30-day, 60-day, and 90-day mortality risk compared to the reference group (Fig 5, S2 Table in S1 File). A U-shaped non-linear relationship was observed between PaCO_2_ levels and 30-day, 60-day, and 90-day mortality risk (*P* for nonlinearity < 0.05) (S1 Fig in S1 File). Additionally, the group with hypocapnia and moderate-to-severe hypercapnia had lower survival probability compared to the normocapnia and mild hypercapnia groups (Log-rank test: *P*<0.0001) (S2 Fig in S1 File).

In the subcohort of SAE patients with GCS>8, it was observed that hypocapnia was associated with higher mortality risk at 30 days, 60 days, and 90 days when compared to the reference group (*P*<0.05), however, the adverse outcomes weren’t observed in the population with hypercapnia (*P*>0.05) (Fig 5, S2 Table in S1 File). Furthermore, The RCS results of subcohort of GCS>8 demonstrated that compared with the other cohorts, there was a L-shaped relationship between PaCO_2_ and the three clinical endpoints rather than U-shaped (S1 Fig in S1 File). This finding aligned with the results obtained from corresponding multivariate logistic regression analyses. It should be noted that the non-linearity test was only statistically significant for the clinical endpoints of 60-day and 90-day (*P*-non-linear<0.05), while it was not significant for the endpoint of 30-day (*P*-overall = 0.014; *P*-non-linear = 0.077). Kaplan-Meier (KM) survival analysis showed that the hypocapnia group had a worse prognosis than the combined group of normocapnia and hypercapnia (Log-rank test: *P*<0.0001) (S2 Fig in S1 File). More detailed methods and data can be found in the S2 Table in S1 File.

## 4. Discussion

SAE is recognized as a prevalent cause of encephalopathy in the intensive care unit [4]. The inflammatory response elicited by sepsis may trigger profound changes in the brain. However, the precise pathogenesis of SAE has not yet been fully understood and may involve neuroinflammatory responses to sepsis, cerebral circulation dysfunction, and disrupted neurotransmission [13]. Although being associated with increased mortality risk and long-term impaired cognition, accurate diagnosis of SAE continues to pose a challenging task to clinicians, and the lack of specific treatment options further compounds the complexity of managing this condition [14]. Therefore, it is imperative for clinicians to prioritize preventive measures, nonspecific treatments, optimal management, and evaluating the impact of some potential treatment options for patients suffering from SAE. To the best of our current understanding, PaCO_2_ is an effective regulator of cerebrovascular tone, modulating cerebral blood flow (CBF) by inducing cerebral vasodilation and constriction [15]. In this retrospective study, we aimed to investigate the relationship between PaCO_2_ and the prognoses of patients suffering from SAE, and to determine the optimal range of PaCO_2_. The main findings of our research are as follows: (1) Patients in the death group had worse physical well-being compared to those in the survival group, including older age, higher severity scores, worse vital signs, more disturbed physiology variables, and more comorbidities; (2) We identified several independent risk factors of 30-day mortality, including age, SOFA, sodium, BUN, PTT, platelet, heart rate, hypocapnia, hypercapnia (moderate and severe), and liver disease; (3) The result of the multivariable logistic analysis revealed that abnormal PaCO_2_ might be associated with 30-day, 60-day, and 90-day mortality; (4) the restricted cube splines regressions indicated a significant nonlinear association between PaCO_2_ and short-term all-cause mortality in patients with SAE; (5) The survival curves showed that normocapnia and mild hypercapnia had a higher survival probability and acquired longer survival time; (6) The results of the relationship between PaCO_2_ and mortality in the matched cohort were consistent with those of the original cohort. (7) Subgroup analysis was conducted by stratifying the original cohort based on the level of disturbance of consciousness. The findings revealed that hypocapnia and moderate-severe hypercapnia were associated with higher 30-day, 60-day, and 90-day mortality in the subcohort of GCS≤8, which aligned with the results obtained from the original cohort. However, in the subcohort of GCS>8, hypocapnia was found to be associated with higher mortality in all three endpoints, rather than any levels of hypercapnia.

The etiology of SAE is complex and likely involves multiple factors. We performed multivariable logistic analyses to identify several independent risk factors for 30-day mortality. Our results showed that age and liver disease, as risk factors, were consistent with previous studies [16, 17]. The SOFA score is a recognized diagnostic criterion for sepsis, proven to be a good indicator of prognosis for critically ill patients [1, 18]. Blood urea nitrogen (BUN) and sodium may reflect renal function. In addition, increased BUN levels were associated with increased mortality of critically ill patients [19]. PTT and platelet count reflect coagulation function, while microcirculation dysfunction is one of the possible mechanisms of SAE. Heart rate is related to cardiac function. Furthermore, propensity score matching (PSM) analysis, as a “post-randomization” statistical method, was performed to strengthen the credibility of multivariate binary logistic regression analysis results. Before and after PSM, the results of the relationship between PaCO_2_ and mortality are consistent.

Hypocapnia and hypercapnia have been implicated in causing secondary brain damage in pathological conditions [20, 21]. Despite this, the underlying mechanism linking PaCO_2_ to mortality risk in SAE remains unclear. We attempted to explore this correlation from a pathophysiological perspective. The normal function of cerebral circulation is crucial for maintaining brain function. However, ischemic brain lesions were observed in histopathologic examinations of SAE patients [4]. Ischemic processes can be classified into two types: macro circulation dysfunction, which includes hypotension, reduced cerebral blood flow, and impaired autoregulation; and microcirculatory impairment, characterized by neurovascular uncoupling, blood-brain barrier dysfunction, and activation of the coagulation cascade [3]. Excessive ventilation, a hallmark of sepsis, can cause hypocapnia (PaCO_2_<35mmHg). Some studies suggest that short-term hypocapnia may be beneficial for some particular conditions, and excessive ventilation could be regarded as a therapeutic intervention by lowing intracranial pressure (ICP) [22–24]. However, this benefit does not appear evident in SAE patients’ prognosis. As mentioned earlier, hypoperfusion is a characteristic of sepsis patients, and vasoconstriction will further decrease cerebral perfusion. Additionally, low PaCO_2_ levels increase the adhesion and aggregation of platelets [20], further impairing microcirculatory function. A retrospective study showed that any levels of hypocapnia were associated with a poor prognosis in patients with brain injury [21], which is consistent with our findings. Controversy about the relationship between hypocapnia and outcomes of neurological disorders has persisted. Furthermore, whether PaCO_2_ affects cerebral autoregulation in SAE patients is still unclear [25–27]. Further basic researches and prospective multicenter clinical studies are necessary to reveal the correlation between low PaCO_2_ and SAE.

Hypercapnia is a condition with increased PaCO_2_ (PaCO_2_≥45mmHg). In our study, patients with hypercapnia were divided into two levels (mild hypercapnia, 45≤PaCO_2_<50mmHg; moderate and severe hypercapnia, PaCO_2_≥50mmHg). In contrast to hypocapnia, hypercapnia increases cerebral blood flow by vasodilation. A study has shown that every 1 mmHg increase in PaCO_2_ can result in a 1–2 ml/100 g/min increase in cerebral blood flow [28]. The prognosis of SAE patients may be influenced differently by hypercapnia depending on their specific general and disease conditions. A retrospective study indicated that hypercapnic acidosis was associated with an increased risk of hospital mortality in patients with cerebral injury, whereas no such association was observed in patients with compensated hypercapnia [29]. In reality, some studies believe that controlled hypercapnia has a neuroprotective effect because of moderately increased cerebral blood flow, while moderate and severe hypercapnia can exacerbate cerebral edema. In addition, cerebral autoregulation would change and the plateau phase of the CBF (cerebral blood flow)-CPP (cerebral perfusion pressure) graph would be shortened. The phenomenon means impaired autoregulation and increased susceptibility of cerebral blood flow to blood pressure fluctuations, which can lead to cerebral ischemia or hemorrhage [15]. For our subgroup study, we found that moderate-severe hypercapnia (PaCO_2_≥50mmHg) was associated with a poor prognosis in the subcohort of SAE patients with severe consciousness disorders. However, there was no significant relationship between any degree of hypercapnia and the risk of mortality in SAE patients with non-severe disorders of consciousness. These findings suggest that the impact of elevated PaCO2 levels may vary depending on the patient’s underlying condition and the severity of the disease. Nevertheless, considering the adverse effects of excessive hypercapnia and the lack of evidence supporting its superiority over normal range, it may be better to maintain normocapnia or controlled mild hypercapnia. It should be noted that the theory of PaCO_2_ regulating CBF is based on healthy populations or patients with brain injury. After conducting a systematic literature search, we found that limited research was performed to investigate the role of PaCO_2_ in autoregulation for patients with SAE. Likewise, further high-quality studies are needed to clarify the correlation between PaCO_2_ levels and mortality risk in SAE patients.

## 5. Limitation

We acknowledge that our study has several limitations. First, it is challenging to diagnose SAE, as mentioned above. The cohort was selected by retrospective analysis in the absence of supporting evidence of image data, which may contribute to information bias. Second, hypercapnia can cause changes in consciousness, which may complicate the selection of study participants. Given the less research examining the relationship between PaCO_2_ levels and mortality in SAE patients, our study provides valuable information despite the limitation. Third, in our study, we just considered short-term all-cause mortality as the clinical endpoints (30-day, 60-day, 90-day), while function outcomes, long-term cognitive impairment and lower quality of life, were ignored because the MIMIC-IV 2.0 database didn’t report them. Fourth, this was an observational study, therefore, the causal relationship between PaCO_2_ and mortality risk cannot be established. Fifth, it should be noted that patients with SAE present a critical and complex condition in the context of sepsis, and there may be potential confounding factors that cannot be eliminated. Finally, our research was a single-center retrospective study and lacked external validation. Therefore, it is essential to perform further large-scale, prospective cohort studies to confirm the findings.

## 6. Conclusion

Among patients suffering from SAE, the initial 24-hour PaCO_2_ levels exhibited a U-shaped relationship with 30-day, 60-day, and 90-day mortality, while the nadirs of the U-curves were within the range of 35–50mmHg. Maintaining the first 24-h PaCO_2_ levels within the normal to mildly elevate (35–50mmHg) was associated with lower mortality risk. The results need to be verified in the prospective trials.

## Supporting information

S1 Data Raw data. (CSV)

S1 File The file contains all supplementary tables and figures. (DOCX)

## Abbreviation

SAE: sepsis-associated encephalopathy
PaCO_2_: Partial Pressure of Carbon Dioxide in Arterial Blood
CBF: cerebral blood flow
GCS: Glasgow Coma Scale
SOFA: Sequential Organ Failure Assessment
BP: blood pressure
SpO_2_: pulse oxygen saturation
BUN: blood urea nitrogen
PTT: partial thromboplastin
MCH: mean corpuscular hemoglobin
WBC: white blood cell
BE: base excess
MI: myocardial infarction
CHF: congestion heart failure
CPD: chronic pulmonary disease
DM: diabetes mellitus
HTN: ypertension
CKD: chronic kidney disease
AKI: acute kidney injury
RRT: renal replacement therapy
