# Supplementary material for: Association between the first 24 hours PaCO2 and all-cause mortality of patients suffering from sepsis-associated encephalopathy after ICU admission: A retrospective study
Source: PLoS One. 2023 Oct 24;18(10):e0293256. doi: 10.1371/journal.pone.0293256 (PMC10597528; doi:10.1371/journal.pone.0293256)

Supplementary Material

# Association between the first 24 hours PaCO_2_ and all-cause mortality of patients suffering from sepsis-associated encephalopathy after ICU admission: a retrospective study

**Honglian Luo^1,2^, Gang Li^2^, Bingxin Yang^1^, Xinlei Huang^2^, Yan Chen^2^***¶**, Wei Shen^1,2^***¶

1. Department of Neurology, Puai Hospital of Tongji Medical College, Huazhong University of Science and Technology, Wuhan, Hubei, 430030, China
2. Wuhan Fourth Hospital, Wuhan, Hubei, 430030, China

*** Correspondence:**

Wei Shen E-mail: [shenwei1971@126.com](mailto:shenwei1971@126.com) (SW) ORCID (0000-0003-2630-9391)

Yan Chen E-mail: 648327266@qq.com

¶: These authors contributed equally to this work.

**S1 Table . Results of the collinearity diagnostic.**

| **Covariates** | **Variance inflation factor** |
| --- | --- |
| Age | 1.576 |
| Gender | 1.132 |
| PaCO_2_ | 1.172 |
| GCS | 1.459 |
| SOFA | 2.758 |
| Temperature | 1.244 |
| Heart Rate | 1.411 |
| Systolic BP | 1.529 |
| Diastolic BP | 1.704 |
| SpO_2_ | 1.207 |
| Glucose | 1.281 |
| Sodium | 3.137 |
| Potassium | 1.190 |
| Bicarbonate | 4.753 |
| Chloride | 4.399 |
| BUN | 2.102 |
| Creatinine | 1.668 |
| PTT | 1.140 |
| Hemoglobin | 1.305 |
| MCH | 1.155 |
| Platelet | 1.341 |
| WBC | 1.116 |
| Lactate | 1.834 |
| BE | 4.802 |
| MI | 1.141 |
| CHF | 1.398 |
| CPD | 1.100 |
| DM | 1.279 |
| HTN | 1.436 |
| CKD | 1.659 |
| Cancer | 1.093 |
| Liver disease | 1.305 |
| AKI(1st) | 1.045 |
| Ventilation(1st) | 1.055 |
| RRT(1st) | 1.365 |
| Vasoactive agent | 1.458 |
| Diuretics | 1.157 |

GCS, Glasgow Coma Scale; SOFA, Sequential Organ Failure Assessment; BP, blood pressure; SpO2, pulse oxygen saturation; BUN, blood urea nitrogen; PTT, partial thromboplastin; MCH, mean corpuscular hemoglobin; WBC, white blood cell; BE, base excess; MI, myocardial infarction; CHF, congestion heart failure; CPD, chronic pulmonary disease; DM, diabetes mellitus; HTN, hypertension; CKD, chronic kidney disease; AKI, acute kidney injury; RRT, renal replacement therapy.

**S2 Table.Two models analysis of the association between PaCO_2_ and all-cause mortality in the subcohorts.**

| Endpoints | The subcohort with GCS≤8 | | | | The subcohort with GCS>8 | | | |
| --- | --- | --- | --- | --- | --- | --- | --- | --- |
|  | Unadjusted model | | Adjusted model | | Unadjusted model | | Adjusted model | |
|  | OR(95%CI) | Pvalue | OR(95%CI) | Pvalue | OR(95%CI) | Pvalue | OR(95%CI) | Pvalue |
| PaCO2 |  |  |  |  |  |  |  |  |
| 35-45mmHg(Ref.) |  |  |  |  |  |  |  |  |
| 30-day mortality |  |  |  |  |  |  |  |  |
| <30 | 2.825(1.908-4.183) | <0.001 | 1.730(1.035-2.893) | 0.037 | 3.029(2.253-4.073) | <0.001 | 1.775(1.216-2.591) | 0.003 |
| 30-35 | 1.844(1.350-2.519) | <0.001 | 1.782(1.211-2.622) | 0.003 | 1.528(1.185-1.970) | 0.001 | 1.428(1.060-1.924) | 0.019 |
| 45-50 | 1.656(1.079-2.541) | 0.021 | 1.439(0.838-2.469) | 0.187 | 1.705(1.208-2.406) | 0.002 | 1.177(0.790-1.752) | 0.423 |
| >50 | 2.993(2.036-4.400) | <0.001 | 1.719(1.013-2.918) | 0.045 | 1.905(1.351-2.688) | <0.001 | 0.861(0.554-1.338) | 0.506 |
| 60-day mortality |  |  |  |  |  |  |  |  |
| <30 | 3.109(2.114-4.573) | <0.001 | 1.926(1.154-3.213) | 0.012 | 3.282(2.505-4.302) | <0.001 | 1.965(1.387-2.784) | <0.001 |
| 30-35 | 1.835(1.356-2.483) | <0.001 | 1.717(1.715-2.509) | 0.005 | 1.554(1.235-1.955) | <0.001 | 1.456(1.110-1.909) | 0.007 |
| 45-50 | 1.529(1.005-2.327) | 0.047 | 1.219(0.711-2.089) | 0.471 | 1.723(1.260-2.356) | 0.001 | 1.245(0.864-1.794) | 0.239 |
| >50 | 3.680(2.516-5.383) | <0.001 | 2.198(1.301-3.714) | 0.003 | 1.846(1.345-2.534) | <0.001 | 0.938(0.625-1.407) | 0.757 |
| 90-day mortality |  |  |  |  |  |  |  |  |
| <30 | 3.085(2.100-4.531) | <0.001 | 1.885(1.126-3.155) | 0.016 | 3.273(2.525-4.243) | <0.001 | 1.926(1.377-2.694) | <0.001 |
| 30-35 | 1.796(1.331-2.423) | <0.001 | 1.632(1.118-2.382) | 0.011 | 1.444(1.159-1.799) | 0.001 | 1.310(1.009-1.700) | 0.043 |
| 45-50 | 1.596(1.057-2.410) | 0.026 | 1.343(0.786-2.293) | 0.281 | 1.640(1.216-2.213) | 0.001 | 1.185(0.833-1.686) | 0.345 |
| >50 | 3.644(2.493-5.327) | <0.001 | 2.231(1.317-3.778) | 0.003 | 1.978(1.473-2.658) | <0.001 | 1.091(0.745-1.597) | 0.655 |

Ref. the range (35-45 mmHg) of PaCO2 was defined as refence (OR=1); Logistic model 1, not adjusted any covariates; logistic model 2, adjusted for the following covariates, including age, gender, GCS, SOFA, sodium, potassium, chloride, BUN, creatinine, hemoglobin, MCH, platelet, WBC, temperature, heart rate, systolic blood pressure, pulse oxygen saturation, and glucose, congestive heart failure, chronic pulmonary disease, diabetes, hypertension, chronic kidney disease, cancer, and liver disease, acute kidney injury, first-day ventilation, first-day RRT, vasoactive medication, and diuretics.Variables related to acid-base balance were omiited because of potential collinearity and MI was excluded because of developing suddenly.

**S1 Fig. Association of the PaCO_2_ and the three clinical endpoints in the subcohort of GCS≤8 (A-C) and the subcohort of GCS>8 (D-E).**


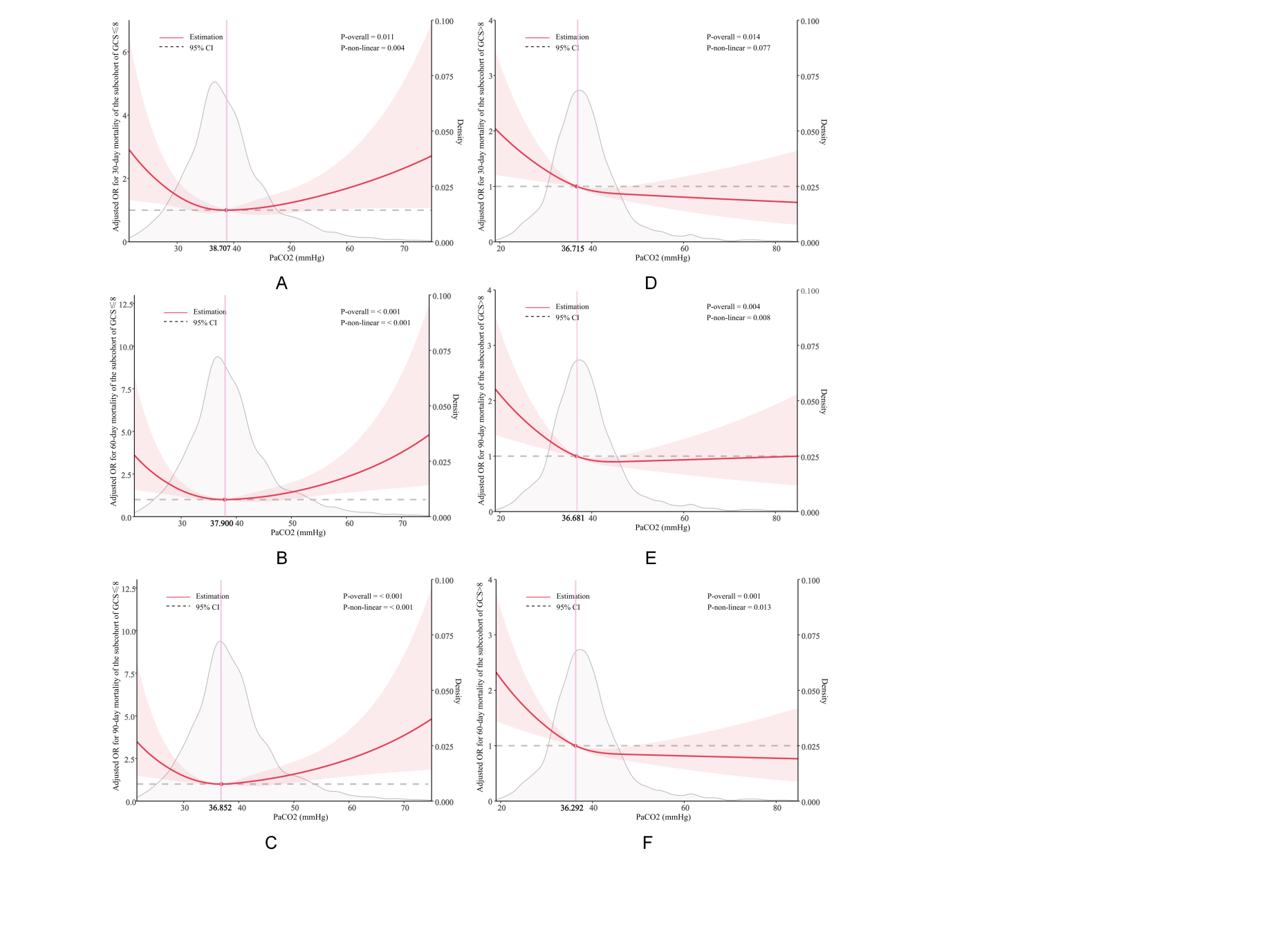


**S2 Fig. Kaplan-Meier survival curves for the subcohorts.**
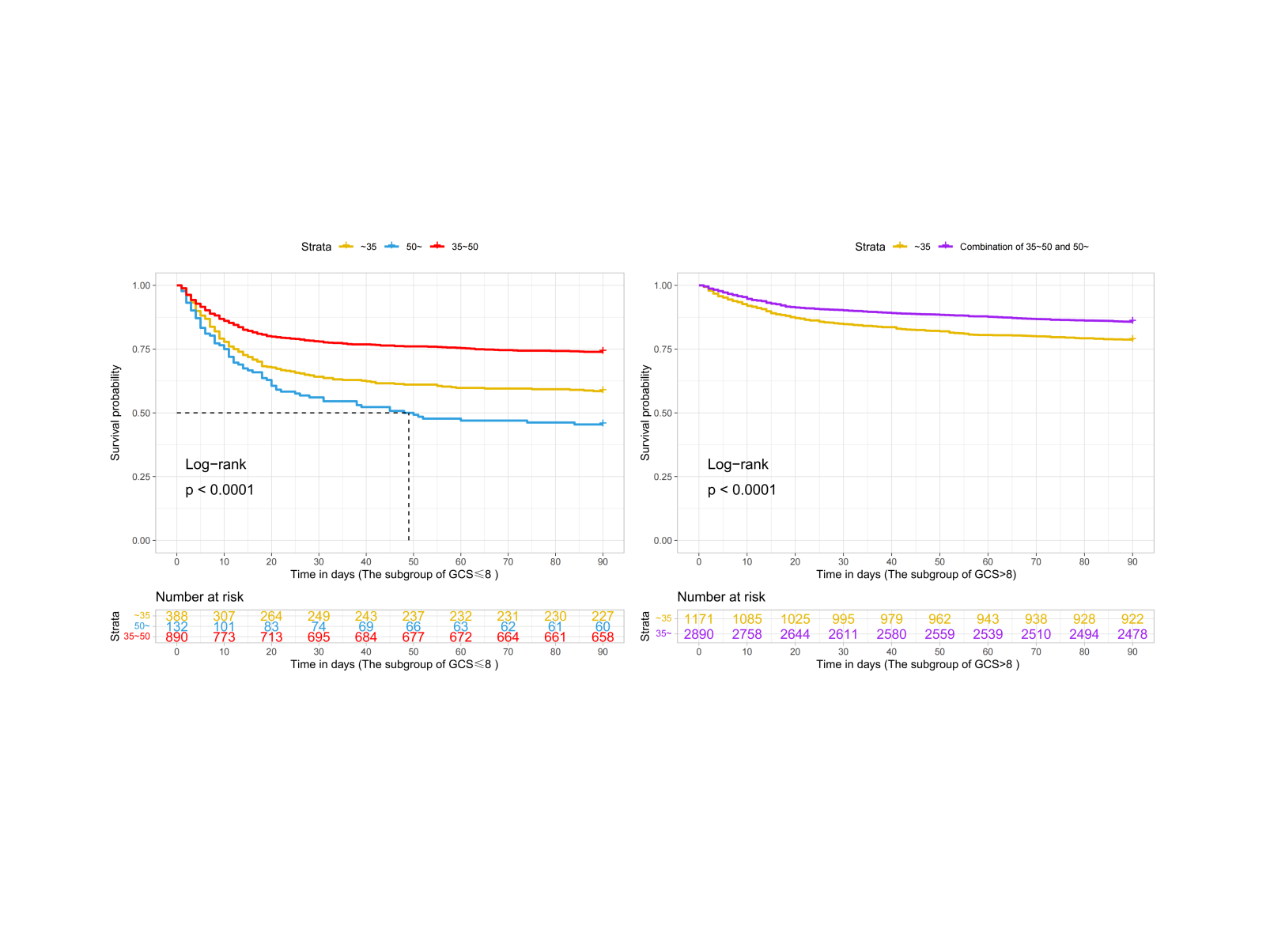

Supplement: S1 File — (DOCX) [file pone.0293256.s002.docx]
